# FDA Risk Assessment of Seafood Contamination after the BP Oil Spill: Rotkin-Ellman and Solomon Respond

**DOI:** 10.1289/ehp.1104539R

**Published:** 2012-02-01

**Authors:** Miriam Rotkin-Ellman, Gina Solomon

**Affiliations:** Natural Resources Defense Council, San Francisco, California, E-mail: mrotkinellman@nrdc.org

We thank Dickey for the opportunity to discuss differing approaches to evaluating health risks from chemical contaminants in food, such as those in Gulf seafood after the BP oil spill disaster. As we demonstrate in our commentary, “Seafood Contamination after the BP Gulf Oil Spill and Risks to Vulnerable Populations: A Critique of the FDA Risk Assessment” (Rotkin-Ellman et al. 2012), the choice of parameters and methods can significantly alter the conclusions of a risk assessment, thereby having major impacts on resulting policy decisions. In the example we analyzed, a risk assessment using parameters and methods specifically aimed at protecting vulnerable populations and incorporating the latest risk science differs from the Food and Drug Administration’s (FDA) approach (FDA 2010) by up to four orders of magnitude.

Dickey characterizes chemical risk assessments as inherently biased “on the side of safety” and is concerned that “unnecessarily conservative risk criteria” could harm human health and society as a whole. This viewpoint, which is arguably rooted more in politics than in science, ignores the long history of chemical assessments where new data and approaches have repeatedly demonstrated significantly greater risks than initially believed (Castorina and Woodruff 2003; Grandjean et al. 2010; Hernberg 2000). Furthermore, from a health-cost perspective, there is considerable benefit to assuring that the population is protected from harmful exposures to carcinogens and other toxicants (Landrigan et al. 2002; Trasande et al. 2006).

A National Research Council (NRC) committee reviewed the status of environmental regulatory risk assessment and concluded that the new science documenting interindividual variability and the vulnerability of the developing fetus and child to chemical contaminants warrants specific changes to risk assessment practices (NRC 2009). These changes were not reflected in the FDA assessment (FDA 2010). The justifications for the FDA’s risk criteria (FDA 2010) that Dickey provides in his letter do not reflect the most current scientific understanding of the health risks from polycyclic aromatic hydrocarbons (PAHs)—or the risk assessment process—and therefore cannot be characterized as biased “on the side of safety.”

The NRC, the U.S. Environmental Protection Agency (EPA), and the broader scientific community have recognized that children are not just small adults and that calculation of life-stage–specific doses are the most health protective method to ensure public health protection (American Academy of Pediatrics 2011; NRC 1993, 2009; U.S. EPA 2005). This necessitates use of age-specific body weights and intake and specifically refutes the claim that an adult body weight and dose can represent risk across a lifespan.

The World Health Organization (WHO) and the U.S. EPA have recognized the extremely skewed nature of food consumption curves and the resulting increased health risk to high-end consumers. These agencies recommended that risk assessments be based on either local surveys (if available) or the 95–97th percentile of national surveys (U.S. EPA 2000; WHO 2008). Previous studies that evaluated the utility of dietary data from the National Health and Nutrition Examination Survey (NHANES) against population-specific surveys have concluded that there is a risk of significantly underestimating exposure among children if NHANES data are the sole source of dietary estimates (Riederer et al. 2010). Furthermore, alternative statistical techniques have been shown to allow better characterization of the upper percentiles in an exposure distribution (Chatterjee et al. 2008). The 90th percentile NHANES dietary values used by the FDA (2010) therefore cannot be characterized as biased toward safety.

The National Toxicology Program (2005) and the California Environmental Protection Agency Office of Environmental Health Hazard Assessment (2005) have determined that there is sufficient evidence to consider naphthalene a carcinogen. The FDA’s reliance on an outdated determination by the U.S. EPA (1998) does not constitute a conservative assessment of the health risks associated with exposures to naphthalene.

Dickey offers the example of the cancer potency factor for benzo(*a*)pyrene (BaP) as specifically demonstrating a “bias toward safety” based on his assertion that it reflects the “95% upper confidence limit of the dose–response curve.” This characterization does not match the description of the cancer potency factor on the Integrated Risk Information System (IRIS) website (U.S. EPA 1994). In fact, the cancer potency factor was based on the “geometric mean of four slope factors obtained by differing modeling procedures” (U.S. EPA 1994). Dickey further asserts that the cancer potency factor “could be as low as zero,” which implies no cancer risk and therefore contradicts the designation of BaP as a carcinogen by multiple authoritative bodies including the FDA (2010), U.S. EPA (1994), Food and Agriculture Organization of the United Nations (FAO)/WHO (2006), and the International Agency for Research on Cancer (IARC 1998).

Last, Dickey cites estimates of annual BaP dietary intake, which he attributes to natural occurrence, as a rationale for not considering the lower acceptable exposure levels we proposed in our commentary (Rotkin-Ellman et al. 2012). Unfortunately this logic is severely flawed and does not comport with the FDA’s charge to protect public health. For an adult, with values based on standard risk assessment methods, the range of total dietary intake Dickey describes (0.16–3.3µg/person/day) corresponds to a lifetime cancer risk ranging from 1.7 × 10^–5^ to 3.4 × 10^–4^—the upper value exceeding what Dickey cites as an acceptable risk range of 1 × 10^–4^ to 1 × 10^–6^. An appropriate FDA response to this finding would be to investigate sources of dietary exposure to PAHs and enact policies to reduce unsafe exposures. This is what the European Union has done in setting standards for BaP in foods of concern (oils and fats, smoked meats, smoked fish, fish, crustaceans, mollusks, baby food, and infant formula) (European Food Safety Authority 2008). To argue that the presence of existing (and potentially unsafe) exposures precludes a thorough assessment of risk for vulnerable populations—because it might identify further risks—runs counter to the tenet of disease prevention inherent in public health protection.

The FDA’s assessments of the risks from contaminants in seafood (e.g., PAHs, mercury), food additives (e.g., bisphenol A, phthalates), and chemicals used in personal care products (e.g., triclosan) have implications for the health of millions of Americans. We hope that our commentary and these letters are the beginning of a fruitful dialogue on how to incorporate advances in the scientific understanding of the impacts of chemical contaminants on vulnerable populations into all risk assessments and policies at the FDA.

## References

[r1] American Academy of Pediatrics (2011). Policy Statement—Chemical-Management Policy: Prioritizing Children’s Health.. Pediatrics.

[r2] Castorina R, Woodruff TJ (2003). Assessment of potential risk levels associated with U.S. Environmental Protection Agency reference values.. Environ Health Perspect.

[r3] Chatterjee A, Horgan G, Theobald C. (2008). Exposure assessment for pesticide intake from multiple food products: a Bayesian latent-variable approach.. Risk Anal.

[r4] European Food Safety Authority 2008 Findings of the EFSA Data Collection on Polycyclic Aromatic Hydrocarbons in Food. EFSA/DATEX/002 http://www.efsa.europa.eu/en/efsajournal/doc/33r.pdf (accessed 7 October 2011).

[r5] FAO/WHO (Food and Agriculture Organization of the United Nations/World Health Organization) (2006). Safety Evaluation of Certain Contaminants in Food. WHO Food Additives Series No. 55.

[r6] FDA (Food and Drug Administration) 2010 Protocol for Interpretation and Use of Sensory Testing and Analytical Chemistry Results for Re-Opening Oil-Impacted Areas Closed to Seafood Harvesting Due to the Deepwater Horizon Oil Spill http://www.fda.gov/food/ucm217601.htm (accessed 7 October 2011).

[r7] Grandjean P, Satoh H, Murata K, Eto K. (2010). Adverse effects of methylmercury: environmental health research implications.. Environ Health Perspect.

[r8] Hernberg S. (2000). Lead poisoning in a historical perspective.. Am J Ind Med.

[r9] IARC (International Agency for Research on Cancer) (1998). Certain Polycyclic Aromatic Hydrocarbons and Heterocyclic Compounds.. IARC Monogr Eval Carcinog Risks Hum.

[r10] Landrigan PJ, Schechter CB, Lipton JM, Fahs MC, Schwartz J (2002). Environmental pollutants and disease in American children: estimates of morbidity, mortality, and costs for lead poisoning, asthma, cancer, and developmental disabilities.. Environ Health Perspect.

[r11] National Toxicology Program (2005). 12th Report on Carcinogens.

[r12] NRC (National Research Council) (1993). Pesticides in the Diet of Infants and Children.

[r13] NRC (National Research Council) (2009). Science and Decisions: Advancing Risk Assessment.

[r14] Office of Environmental Health Hazard Assessment (2005). No Significant Risk Level (NSRL) for the Proposition 65 Carcinogen Naphthalene. Sacramento, CA:Office of Environmental Health Hazard Assessment, California Environmental Protection Agency.. http://oehha.ca.gov/prop65/law/pdf_zip/Naphthalene_NSRL_2005at%20.pdf.

[r15] Riederer AM, Pearson MA, Lu C (2010). Comparison of food consumption frequencies among NHANES and CPES children: implications for dietary pesticide exposure and risk assessment.. J Expo Sci Environ Epidemiol.

[r16] Rotkin-Ellman M, Wong KK, Solomon GM (2012). Seafood contamination after the BP Gulf oil spill and risks to vulnerable populations: a critique of the FDA risk assessment.. Environ Health Perspect.

[r17] Trasande L, Schechter C, Haynes KA, Landrigan PJ (2006). Applying cost analyses to drive policy that protects children: mercury as a case study.. Ann NY Acad Sci.

[r18] U.S. EPA (U.S. Environmental Protection Agency) (1994). Integrated Risk Information System: Benzo[a]pyrene (BaP) (CASRN 50-32-8).. http://www.epa.gov/iris/subst/0136.htm.

[r19] U.S. EPA (U.S. Environmental Protection Agency) (1998). Integrated Risk Information System: Naphthalene (CAS No. 91-20-3).. http://www.epa.gov/iris/subst/0436.htm.

[r20] U.S. EPA (U.S. Environmental Protection Agency) (2000). National Guidance: Guidance for Assessing Chemical Contaminant Data for Use in Fish Advisories. Volume 2. Risk Assessment and Fish Consumption Limits—Third Edition. EPA 823-B-00-007.. http://water.epa.gov/scitech/swguidance/fishshellfish/techguidance/risk/volume2_index.cfm.

[r21] U.S. EPA (U.S. Environmental Protection Agency) (2005). Supplemental Guidance for Assessing Susceptibility from Early-life Exposure to Carcinogens. EPA/630/R-03/003F.

[r22] WHO (World Health Organization) (2008). Highest Reported 97.5th Percentile Consumption Figures (Eaters Only) for Various Commodities by the General Population and Children Ages 6 and Under. GEMS/Food for the Codex Committee on Pesticide Residues and the Joint FAO/WHO Meetings on Pesticide Residues.. http://www.who.int/foodsafety/chem/en/acute_hazard_db1.pdf.

